# Sensitive methods for detection of the S768R substitution in exon 18 of the *DDR2* gene in patients with central nervous system metastases of non-small cell lung cancer

**DOI:** 10.1007/s12032-014-0176-4

**Published:** 2014-08-31

**Authors:** Marcin Nicoś, Tomasz Powrózek, Paweł Krawczyk, Bożena Jarosz, Beata Pająk, Marek Sawicki, Krzysztof Kucharczyk, Tomasz Trojanowski, Janusz Milanowski

**Affiliations:** 1 Department of Pneumonology, Oncology and Allergology, Medical University of Lublin, Jaczewskiego 8, 20-954 Lublin, Poland; 2 Postgraduate School of Molecular Medicine, Medical University of Warsaw, 02-091 Warsaw, Poland; 3 Pathological Laboratory, Department of Neurosurgery and Pediatric Neurosurgery, Medical University of Lublin, 20-954 Lublin, Poland; 4 BioVectis Ltd, Kucharczyk TE, 02-106 Warsaw, Poland; 5 Electron Microscopy Platform, Mossakowski Medical Research Centre, Polish Academy of Sciences, 02-106 Warsaw, Poland; 6 Department of Thoracic Surgery, Medical University of Lublin, 20-954 Lublin, Poland; 7 Institute of Agricultural Medicine, 20-090 Lublin, Poland

**Keywords:** *DDR2* mutation, NSCLC, Central nervous system metastases

## Abstract

Discoidin death receptor 2 (DDR2) receptor belongs to a DDR family that shows a tyrosine kinase activity. The somatic mutations in *DDR2* gene, reported in non-small cell lung cancer (NSCLC), are involved in up-regulation of cells’ migration, proliferation and survival. A S768R substitution in *DDR2* gene was commonly reported in squamous cell lung carcinoma. Clinical data of patients carrying the *DDR2* gene mutation suggest that its presence can be independent of gender and age. The effectiveness of an oral dual-specific (Src and Abl) multikinase inhibitors—dasatinib—was observed in different cell lines and in some NSCLC patients with identified *DDR2* mutation. In the present study, we have used three molecular methods (ASP-real-time PCR, ASP-DNA-FLA PCR and direct sequencing) to detect the *DDR2* gene mutation in 143 patients with NSCLC metastases to the central nervous system (CNS). The prevalence of the *DDR2* gene mutation was correlated with the occurrence of mutations in the *EGFR*, *KRAS*, *HER2* and *BRAF* genes. We identified three patients (2.1 % of studied group) with *DDR2* mutation. The mutation was observed in two patients with low differentiated squamous cell lung cancer and in one patient with adeno-squamous cell carcinoma (ADSCC). In ADSCC patients, *DDR2* mutation coexisted with G12C substitution in *KRAS* gene. According to the current knowledge, examination of the presence of the *DDR2* gene mutation in metastatic lesion is the first such report worldwide. The information, that these driver mutations are present in CNS metastases of NSCLC, could broaden therapeutic choices in such group of patients.

## Introduction

Discoidin death receptor 2 (DDR2) receptor belongs to a DDR family that shows tyrosine kinase activity. A collagen is needed to stimulate phosphorylation of the DDR2 receptor that promotes cell migration, proliferation and survival. Apart from its important role in development and tissue repair, the DDR2 also regulates primary and metastatic cancer progression by down-streaming of the DDR2 signaling pathways including Shp-2, Src, STAT and MAPK. Rare frequency of the *DDR2* gene mutations has been reported in kidney, breast, brain, endometrial and colon cancers [1, 2]. However, a substitution of serine to arginine at position 768 in exon 18 of the *DDR2* (S768R) gene has been most commonly described (2.2–3.8 %) in squamous cell lung carcinoma (SCC) patients and in smokers. No alterations in the *DDR2* gene copy number or protein over-expression were reported [3, 4].

The clinical data of patients with *DDR2* gene mutation suggest that its presence is independent of gender and age. A functional characteristic has showed that cells carrying this mutation are sensitive to an oral dual-specific (Src and Abl) multikinase inhibitors (dasatinib, imatinib, nilotinib, ponatinib, sorafenib and pazopanib) [2, 4]. It also had been previously mentioned that dasatinib inhibited lung cancer cell lines with *DDR2* mutation [5, 6]. A phase II trial (NCT00787267) determined the dasatinib activity in previously treated patients with advanced non-small cell lung cancer (NSCLC). However, another phase II study (NCT01514864) recruited only SCC patients with *DDR2* mutation and indicated satisfactory results of the dasatinib as first- or subsequent-line therapy in this group of patient [4, 5, 7].

In the future, the presence of the *DDR2* gene mutation may become a potential predictive marker for the effectiveness of molecularly targeted therapies in SCC patients group. However, there is no evidence on the prevalence of the *DDR2* gene mutations in a metastatic NSCLC. Taking into account that the central nervous system (CNS) is one of the most frequent location for metastases of NSCLC, the aim of the study was to estimate the presence of the S768R substitution in the *DDR2* gene in tissue samples from Caucasian patients with the CNS metastases of NSCLC.

## Materials and methods

### Patients and material

A total of 143 Caucasian patients with NSCLC metastatic lesions in the CNS were enrolled in present retrospective study. The corresponding primary NSCLC tumors were simultaneously available from 30 patients. The patients underwent routine neurosurgical procedures with a palliative aim, and the median survival time from lung cancer diagnosis to death was 9.2 months. Moreover, none of patients was treated with chemotherapy, radiotherapy or molecularly targeted therapies. Detailed characteristic of studied group has been presented in Table 1.

**Table 1:** Characteristic of studied group

*Gender*	
Male, *n* (%)	99 (69.2)
Female, *n* (%)	44 (30.8)
*Age*	
Median age ± SD (years)	60 ± 8.8
≥60 years, *n* (%)	71 (49.7)
<60 years, *n* (%)	72 (50.3)
*Pathomorphological diagnosis*	
Adenocarcinoma, *n* (%)	61 (42.7)
Squamous cell carcinoma, *n* (%)	23 (16.1)
NSCLC-NOS, *n* (%)	38 (26.6)
Large cell carcinoma, *n* (%)	21 (14.6)
*Smoking status*	
Smokers, *n* (%)	86 (60.1)
Non-smokers, *n* (%)	32 (22.4)
Lack of data, *n* (%)	25 (17.5)

The study was approved by the Ethics Committee of the Medical University of Lublin, Poland (No. KE-0254/86/2013).

### Mutation analysis

DNA was isolated from formalin-fixed paraffin-embedded (FFPE) metastatic tissue samples using QIAamp DNA FFPE tissue kit (Qiagen, USA) according to a manufacturer’s protocol. Estimation of the S768R mutation in the *DDR2* gene was conducted using three methods based on a PCR: the allele-specific real-time PCR (ASP-real-time PCR), the allele-specific PCR with DNA fragment length analysis (ASP-DNA-FLA PCR) and the direct sequencing. Additionally, the incidence of mutation in *EGFR* (deletions in exon 19 and substitutions: L858R, T790M, L861Q, S768I, G719X), *HER2* (A775YVMA or M774AYMVM insertion), *KRAS* (codon 12, 13 and 61) and *BRAF* (V600E substitution) genes also was assessed in the analyzed material.

### ASP-real-time PCR method

The S768R substitution was analyzed using the real-time PCR method with DNA intercalating dye and the allele-specific primers for mutated (mt) and wild-type (wt) *DDR2* gene. Total volume of PCR mixture (15 µl) contains: 8 µl of Master Mix (PrimerDesign Ltd, UK) with DNA intercalating dye (Chromofy™), 1 µl of purified genomic DNA (20 ng/µl), 2 µl of each primers for mt or wt *DDR2* gene and 4 µl of nuclease-free water. The amplification of examined region was performed in 48-well plates using the Eco real-time PCR device (Illumina, USA) in following steps: pre-denaturation 95 °C-10 min and 35 cycles in conditions: 95 °C-15 s and 62 °C-60 s. The codon 768 of *DDR2* gene was tested simultaneously in each sample for mt and wt form in the same PCR. The negative control of the ASP-real-time PCR was the reaction with DNA isolated from peripheral blood leukocytes of healthy individual. Samples were assessed as positive if amplification in the real-time PCR was observed both for mutant and for wild type of *DDR2* gene. The samples with late amplification (*C*_t_ > 32 cycle) of wt region of codon 768 were excluded from analysis, and samples with late amplification (*C*_t_ > 32 cycle) of mt region of *DDR2* gene were assessed as negative.

### ASP-DNA-FLA PCR analysis

The S768R substitution was analyzed using a PCR with the allele-specific primers. Sequences of primers were the same as in the real-time PCR, but one of the primers was labeled by fluorochrome Cy5. The PCR mixture contained: 10 µl of Master Mix (Thermo Scientific, USA), 1 µl of purified genomic DNA (20 ng/µl), 2 µl of each primers for mt or wt of *DDR2* and nuclease-free water up to 20 µl of total volume. PCR was performed in TPersonal thermocycler (Biometra, Germany) in following steps: pre-denaturation (95 °C-10 min), 35 cycles (95 °C-30 s, 64 °C-40 s and 72 °C-45 s) and ended elongation (72 °C-10 min). The length analysis of the amplified DNA fragments in polyacrylamide gel (DNA-FLA) was conducted in ALF Express II sequencer using ALFwin Fragment Analyzer (Amersham Pharmacia Biotech, Sweden). The product of amplification was observed in length of 129 bp. DNA isolated from peripheral blood leukocytes of healthy individual was used as a negative control. The samples were assessed as positive if amplification was observed as peaks both for mt and for wt of the *DDR2* gene in ALFwin Software. The samples with a low fluorescence level (RFU < 10) for wild type of codon 768 were excluded from analysis and were repeated in next PCR. The samples with a low fluorescence level (RFU < 10) for mutated codon 768 were assessed as wild type.

### Direct sequencing

The Sanger cycle sequencing reactions were performed in separate vials for each dideoxynucleotide. 100 µg of the PCR products were purified with ExoSap enzyme mix (according to the manufacturer’s instructions) and used as template. The sequencing was carried out with Big Dye Terminator v3.1 Cycle Sequencing RR-2500 according to the manufactures instructions. The cycle sequencing conditions for the PCR product as template were as follows: 95 °C for 10 s, 52 °C for 5 s and 60 °C for 3 min for 75 cycles.

### Calculation of mutated DNA content in examined samples and statistical analysis

During the next part of the study, we used mathematical formula to estimate the percentage content of mt DNA in the samples with detected mutation. The mutation level for amplified region was determined according to the following equation:

$$\% {\text{ mutated DNA}} = 2^{{ - \varDelta C_{\text{t}} }} \times 100 \%$$

*ΔC*_t_ (analyzed sample) = the average *C*_t_ value from the mutant reaction − the average *C*_t_ value from the wild-type reaction.

The data were present as a frequency of *DDR2* gene mutation in analyzed group and subgroup of patients.

## Results

**Fig. 1:**
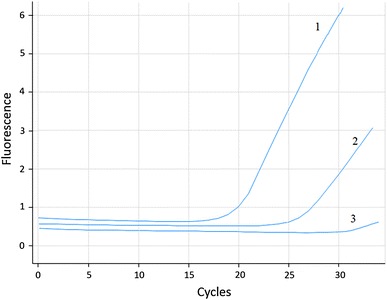
Amplification curves of *DDR2* gene in ASP-real-time PCR analysis. Curve *1* represents amplification of wt *DDR2* region and curve *2* amplification of product for mt *DDR2* region in the same patient. Curve *3* represents negative control of reaction

Based on the real-time PCR results, the presence of amplified region for the S768R substitution in the *DDR2* gene was revealed (mean *C*_t_ = 25th cycle) in four patients (5.6 % of evaluated group, Fig. 1) with the CNS metastases of NSCLC. In each case, the amplification of wt control also was also observed (mean *C*_t_ = 20th cycle), as well as the presence of mt amplification in negative control was not shown. In patients with the *DDR2* mutations, corresponding primary tumor was not available.

**Fig. 2:**
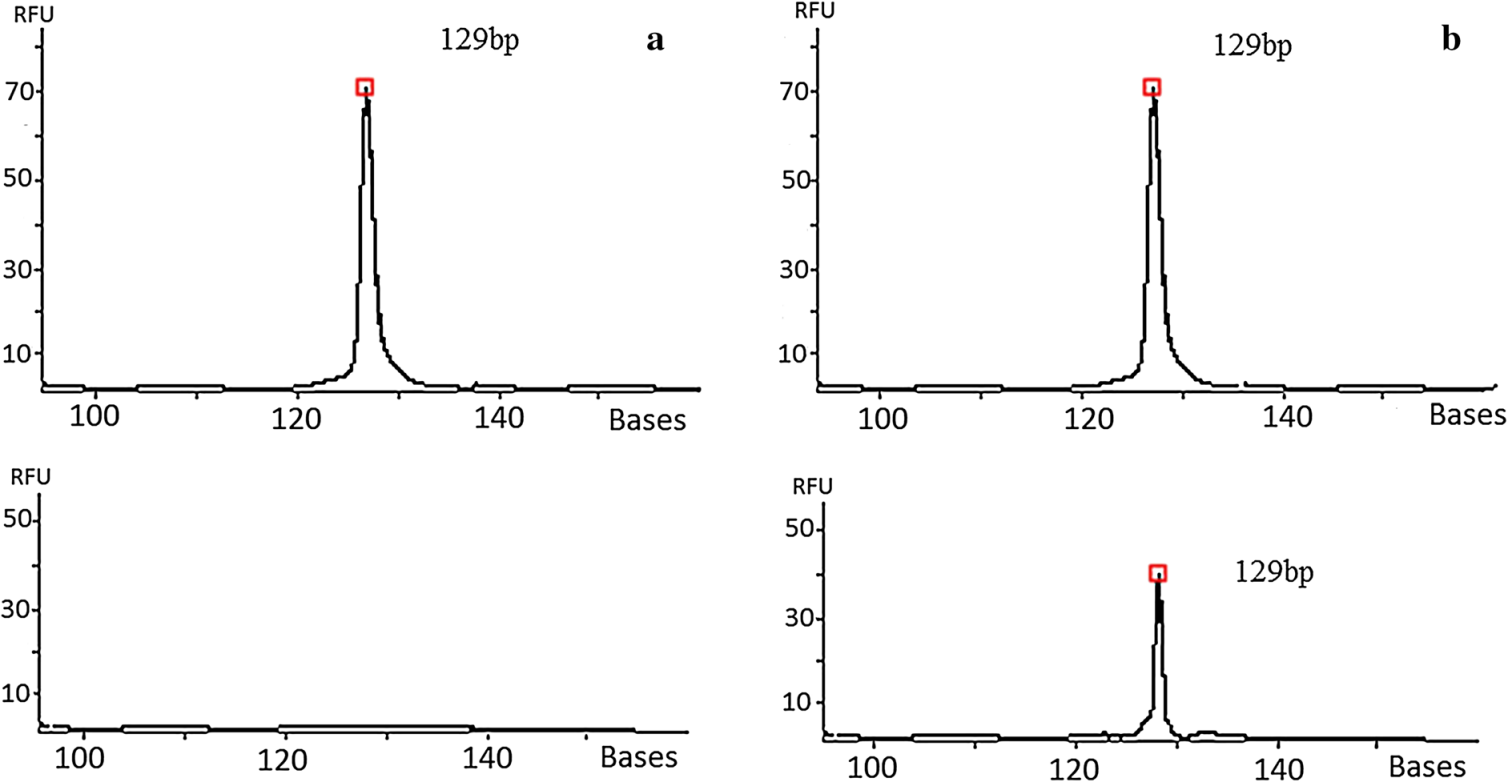
Example of *DDR2* gene examination in ASP-PCR-DNA-FLA. **a** The presence of *DDR2* amplification with primer complementary to wt of *DDR2* gene and lack of *DDR2* amplification with a primer complementary to the mt *DDR2* gene. **b** The presence of *DDR2* amplification both with primer complementary to wt and mt *DDR2* gene

In second part of the study, all the real-time PCR positive samples were tested for the S768R substitution in ASP-DNA-FLA PCR and in the direct sequencing. However, only in three samples, the product of amplification (Fig. 2b) was observed during the ASP-DNA-FLA PCR assay. The product of amplification was observed in patients with 7.5–9 % of mutated DNA. However, the ASP-DNA-FLA PCR was negative in one patient with low content of mutated DNA (<4 %). Therefore, there is a probability that in this case the result of the real-time PCR was false-positive caused by nonspecific binding of primers or the sensitivity of ASP-DNA-FLA PCR was too low.

Unfortunately, the direct sequencing only showed the presence of wt *DDR2* gene in all analyzed samples. However, based on mathematical analysis, the content of mt DNA in all positive samples was assessed as <10 % (Table 2), making it impossible to obtain reliable results in direct sequencing method. Moreover, the low quality of DNA isolated from FFPE tissue samples and sub-clonality of DDR2 mutations in the metastases could have affected on the results of direct sequencing analysis. All three methods have different sensitivity of mt DNA detection that had been previously described (direct sequencing >40 %, real-time PCR >1 %, ASP-DNA-FLA PCR >5 %) [8–10]. Taking into account a low purity of the analyzed samples, the next generation sequencing method had not been used to verify positive results.

**Table 2:** Results of analysis of *DDR2* gene according to the type of methods used for detection of *DDR2* gene abnormalities

Patients (% of mt DNA)	Pathomorphological diagnosis	Methods (sensitivity)		
		Real-time PCR (>1 % of mt DNA)	ASP-DNA-FLA PCR (>5 % of mt DNA)	Direct sequencing (>10 % of mt DNA)
1 (9 %)	SCC	+	+	−
2 (8 %)	SCC	+	+	−
3 (7.5 %)	ADSCC	+	+	−
4 (4 %)	AC	+	−	−

According to real-time PCR and ASP-PCR-DNA-FLA analysis, we detected 3 patients (2.1 % of evaluated group) with S768R substitution in *DDR2* gene. All three *DDR2* gene-positive patients were current or former smokers. Two patients (52-year-old male and 60-year-old female) were diagnosed SCC (8.7 % of SCC), and their *DDR2* gene mutation was mutually exclusive. Moreover, one 70-year-old female was diagnosed adeno-squamous cell lung carcinoma based on microscopy examination and immunohistochemistry (IHC) staining and she also was carrier of G12C substitution in *KRAS* gene.

Analysis of *EGFR*, *KRAS*, *HER2* and *BRAF* mutations did not show any coexistence with S768R mutation in *DDR2* gene, besides one mentioned patient.

Additionally, in previous studies, we reported detection of 9 (6.29 % of studied group) common activating *EGFR* gene mutations (6 L858R substitutions and 3 deletion in exon 19), primary T790 M mutation in 3 (2.1 %) patients, 20 (14 %) *KRAS* gene mutations (19 in codon 12 and 1 in codon 61), 1 (0.67 %) insertion in *HER2* gene and none V600E substitution in *BRAF* gene.

## Discussion

Our findings presented in this study have shown that the S768R substitution in the *DDR2* gene is detectable not only in patients with primary NSCLC but also in metastatic lesions of lung cancer. According to current knowledge, our study about the presence of the *DDR2* gene mutation in a metastatic lesion is the first such report worldwide. The prevalence of these mutations (2.1 %) in the CNS metastases of NSCLC in Caucasian patients is in accordance with previous studies provided in primary tumors [3, 11, 12]. Moreover, the *DDR2* gene mutation is commonly detected in SCC primary tumors [2, 3, 12]. Two of our patients were also diagnosed such type of lung cancer. However, one patient was diagnosed low differentiated adeno-squamous lung carcinoma and she also was carrying both of *DDR2* and *KRAS* gene mutations. It is possible that the *DDR2* gene mutation was detected in squamous cell component of lung cancer and *KRAS* gene mutation was detected in adenocarcinoma component of this malignancy. However, the coexistence between driver mutations was seldom reported, and microdissection was not performed to confirm this suspicion [13, 14].

Despite of the low sensitivity, the direct DNA sequencing is still considered as gold standard for mutation detection. However, it was indicated that techniques based on the real-time PCR methodology (for example, PNA-mediated real-time PCR clamping) are highly sensitive and allow to detect mutant alleles even at 100-fold lower levels than wild type [8, 15, 16]. On the other hand, the direct sequencing allowed to detect two uncommon not activated mutations. Moreover, several authors suggested that to improve the sensitivity of molecular analysis all tissue samples should be evaluated in the microscope by a pathologist to select an appropriate area with high proportion of tumor cells [8, 16].

For the first time, somatic mutations in *DDR1* and *DDR2* genes were reported by the Davis et al. [11] in two lung cancer patients (one SCC and one large cell carcinoma) and in one lung cancer cell line (NCI-H1770). On the other hand, the Ford et al. demonstrated that the expression of DDRs receptor is significantly deregulated in NSCLC tumors. Furthermore, the Ford et al. [12] suggested that the genetic status of *DDR1* and *DDR2* genes can be an independent favorable prognostic marker for early stages of NSCLC.

Hammerman et al. [3] had sequenced 290 SCC tissue samples and indicated 11 (3.8 %) *DDR2* gene mutations that were found both in the sequence of a kinase domain and in other regions of this gene. Apart from nine common S768R substitutions, they identified two rare G774 mutations. Moreover, they found L239R and I638F mutations in the HCC-366 and NCI-H2286 SCC cell lines, respectively. The analysis of the *DDR2* gene copy number did not reveal the DDR2 over-expression in tested group. Unfortunately, clinical data of the patients were limited; therefore, the presence of *DDR2* gene mutation did not show any correlation with age, gender or smoking status.

Johnson et al. [7] on the phase II study demonstrated clinical activity of the dasatinib in a molecularly unselected population of patients with NSCLC. However, the response rate was not favorable in comparison with standard therapy response rate (43 vs. 40 % of disease control rates). Progression-free survival of examined patients was 1.36 months in the dasatinib treatment arm and 3.6 months in control arm. For this reason, they suggested that future studies of the dasatinib should be based on molecular predictive factors.

It has been shown that tumor cells with oncogenic *DDR2* gene mutation can be effectively inhibited by multikinase inhibitors [2, 4, 5, 7]. Hammerman et al. [3] had reported that the dasatinib showed particular efficiency against SCC cell lines bearing *DDR2* gene mutations. The dasatinib inhibited proliferation of the *DDR2* mutant NCI-H2286 and HCC-366 cells while the imatinib was less effective in the same tested cell lines. Moreover, the dasatinib and the imatinib were less effective against the A549 cell line, which carries a *KRAS* mutation and does not have any *DDR2* mutations. On the other hand, the NCI-H1703 SCC cell line, which contained a *PDGFRA* amplification, was sensitive to both drugs.

Pitini et al. [17] presented a case report of 50-year-old women, all heavy smokers, with coexistence of primary SCC and chronic myelogenous leukemia that was treated with the dasatinib. After 10 weeks of the dasatinib therapy, the patient had normal blood counts and the lung tumor was nearly completely resolved. Eight months after starting the treatment, when patients still responded to the dasatinib, authors sequenced kinase domain of *DDR2* gene and identified the S768R mutation. This result confirmed the suspicions that the dasatinib can effectively inhibit the growth of tumor with *DDR2* gene mutation.

Based on the overall data, we would like to conclude that the *DDR2* gene mutation is detectable in the CNS metastases of NSCLC, and analysis of gene profile in cancer patients may extend the scope of molecularly targeted therapies used both in patients with primary and metastatic NSCLC. Moreover, in the near future, the personalized therapy based on the assessment of different gene mutations in NSCLC patients may become a reality.

## Abbreviations

ADSCC: Adeno-squamous cell carcinoma
ASP-PCR: Allele-specific polymerase chain reaction
CNS: Central nervous system
DDR2: Discoidin death receptor 2
FFPE: Formalin-fixed paraffin-embedded
FLA: Fragment length analysis
NSCLC: Non-small cell lung cancer
SCC: Squamous cell lung carcinoma
